# Supplementary figures and images for: Multiple modes of cholesterol translocation in the human Smoothened receptor
Source: eLife. 2026 Mar 11;14:RP108030. doi: 10.7554/eLife.108030 (PMC12978703; doi:10.7554/eLife.108030)

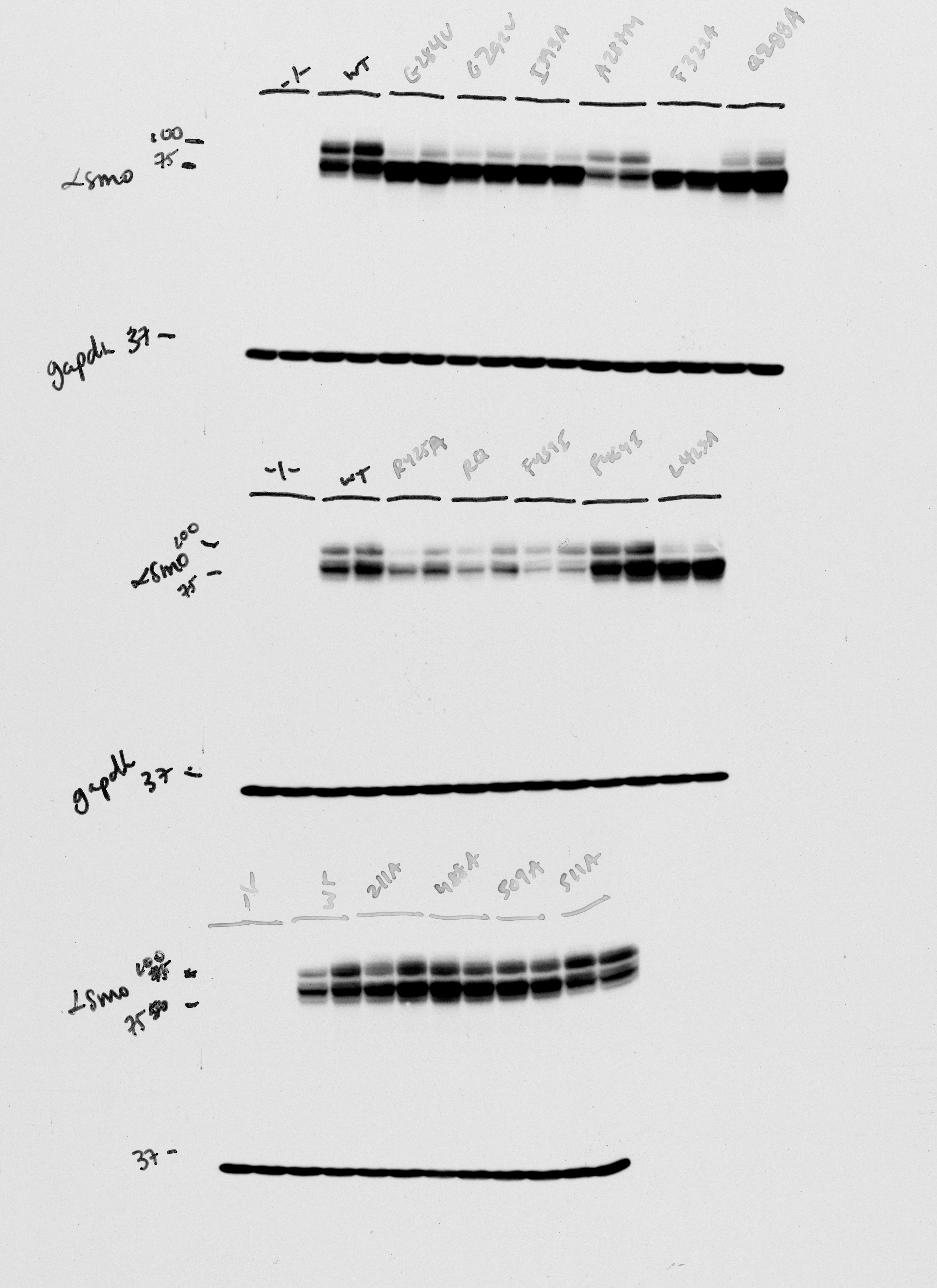

Supplement: Figure 3—figure supplement 1—source data 2. [file elife-108030-fig3-figsupp1-data2.zip › Fig3_FigSupp1a_original_images/smo-MD-Prateek-pathways20240328_09134266_0002.jpg]

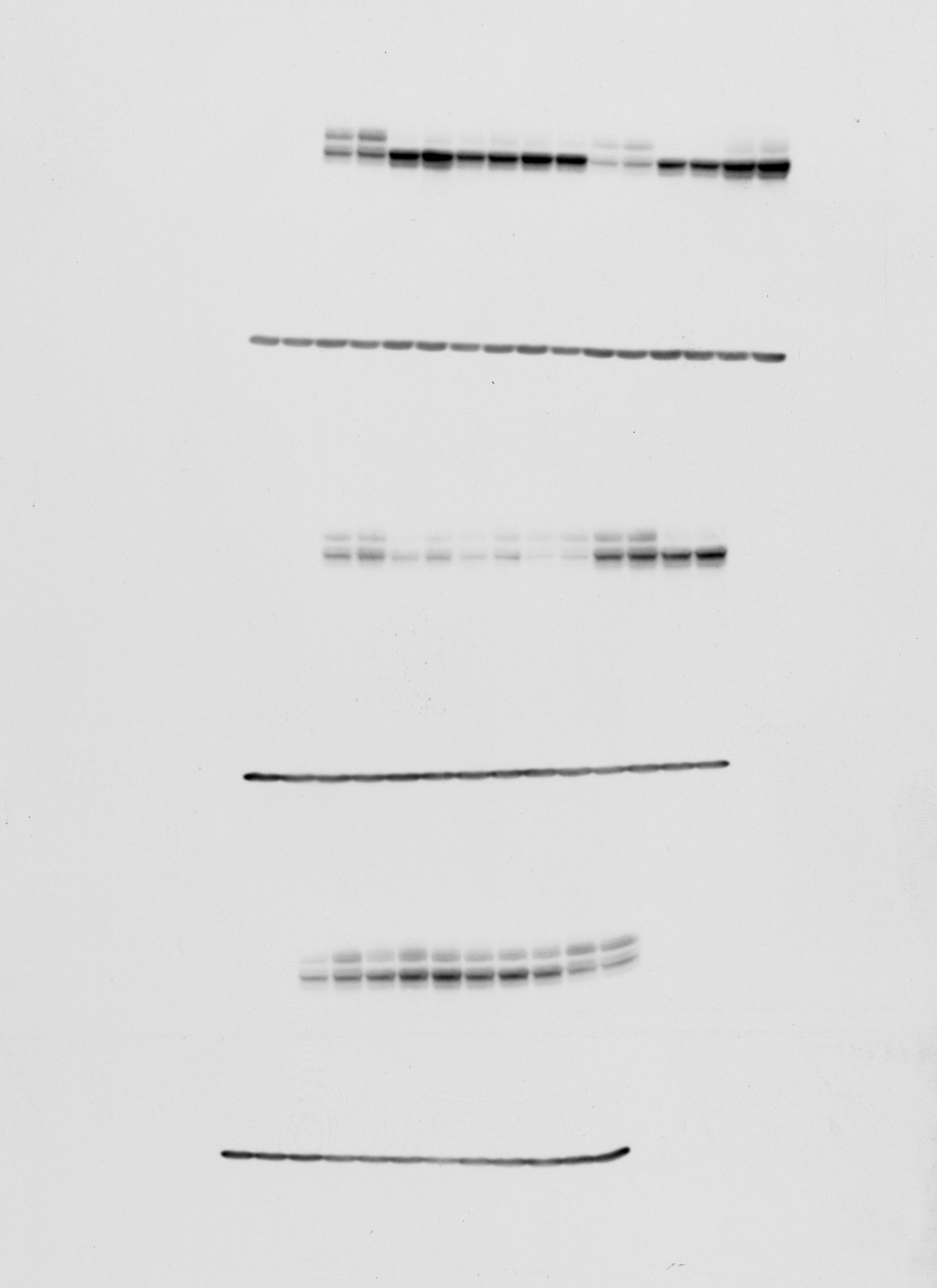

Supplement: Figure 3—figure supplement 1—source data 2. [file elife-108030-fig3-figsupp1-data2.zip › Fig3_FigSupp1a_original_images/smo-MD-Prateek-pathways20240328_09122913_0001.jpg]

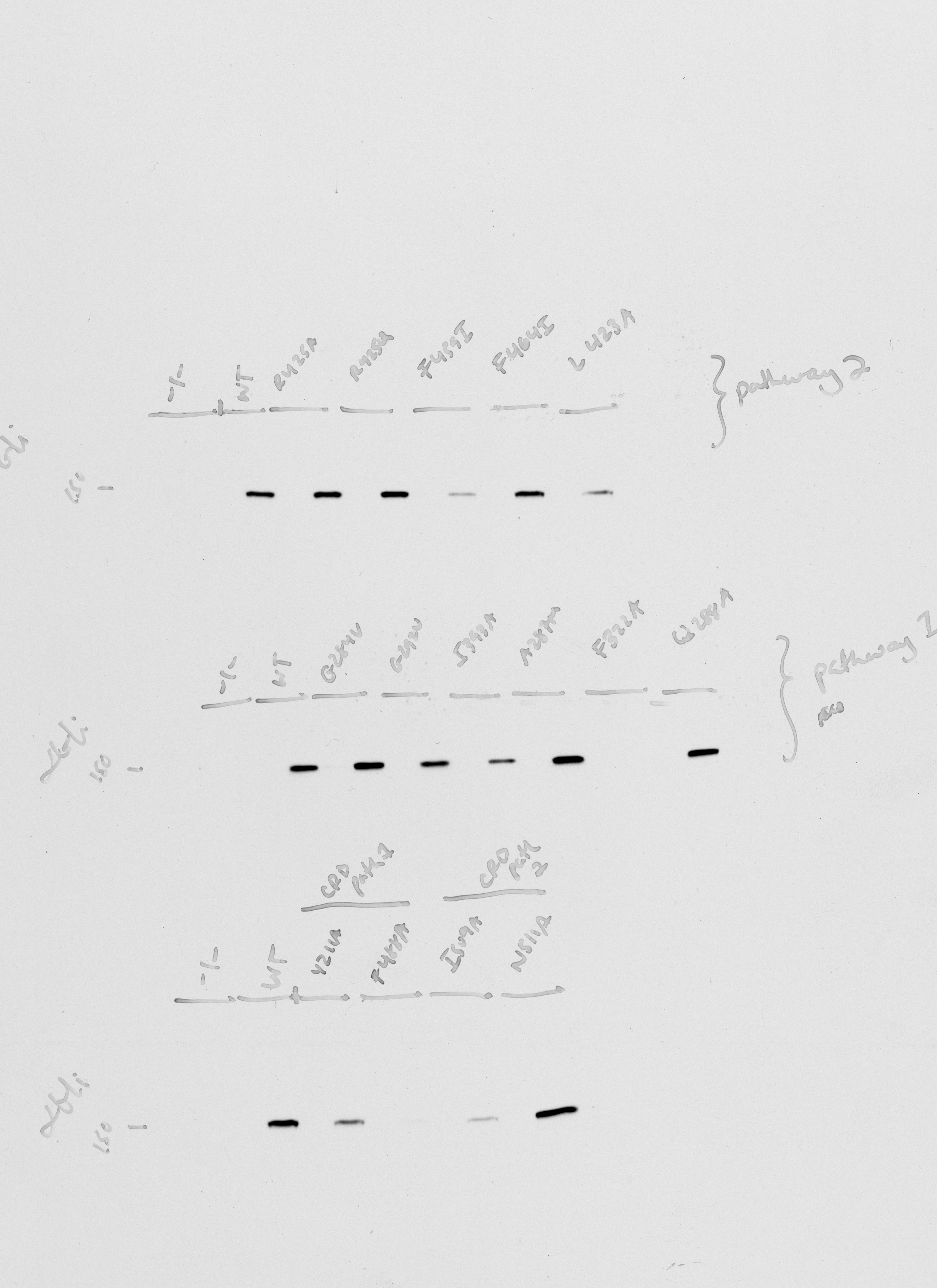

Supplement: Figure 3—figure supplement 1—source data 2. [file elife-108030-fig3-figsupp1-data2.zip › Fig3_FigSupp1a_original_images/smo-MD-Prateek-pathways20240328_09150106_0003.jpg]
