# Supplementary material for: Multiple modes of cholesterol translocation in the human Smoothened receptor
Source: eLife. 2026 Mar 11;14:RP108030. doi: 10.7554/eLife.108030 (PMC12978703; doi:10.7554/eLife.108030)

Related to Figure 3- figure supplement 1

Data depicted in figure is shown in red dashed boxes

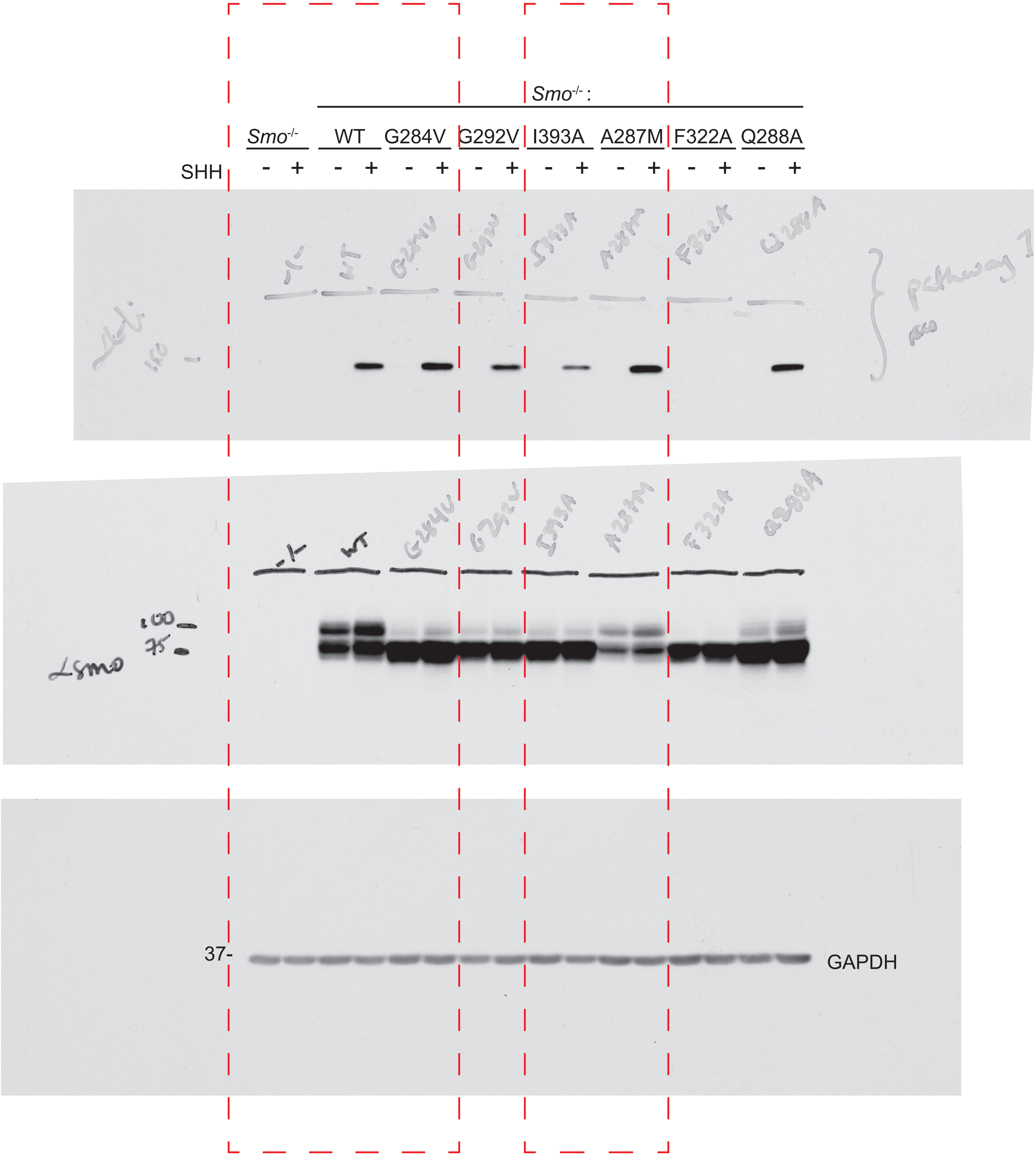

Related to Figure 3- figure supplement 4

Data depicted in figure is shown in red dashed boxes

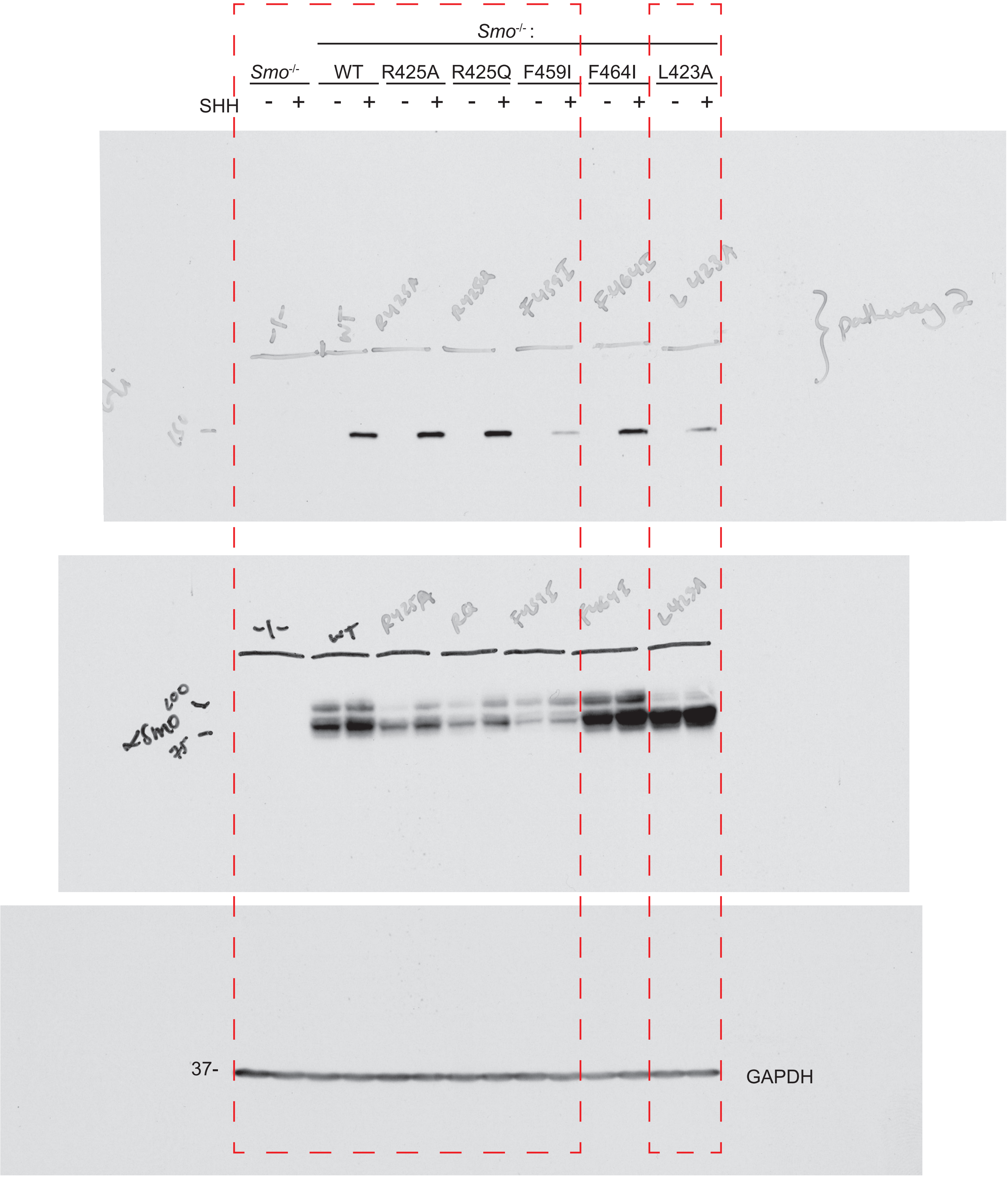

Related to Figure 6- figure supplement 1

Data depicted in figure is shown in red dashed boxes

*I509A is the human numbering and was mislabeled on this blot. The mouse numbering is I5113A, which is corrected in the figure.

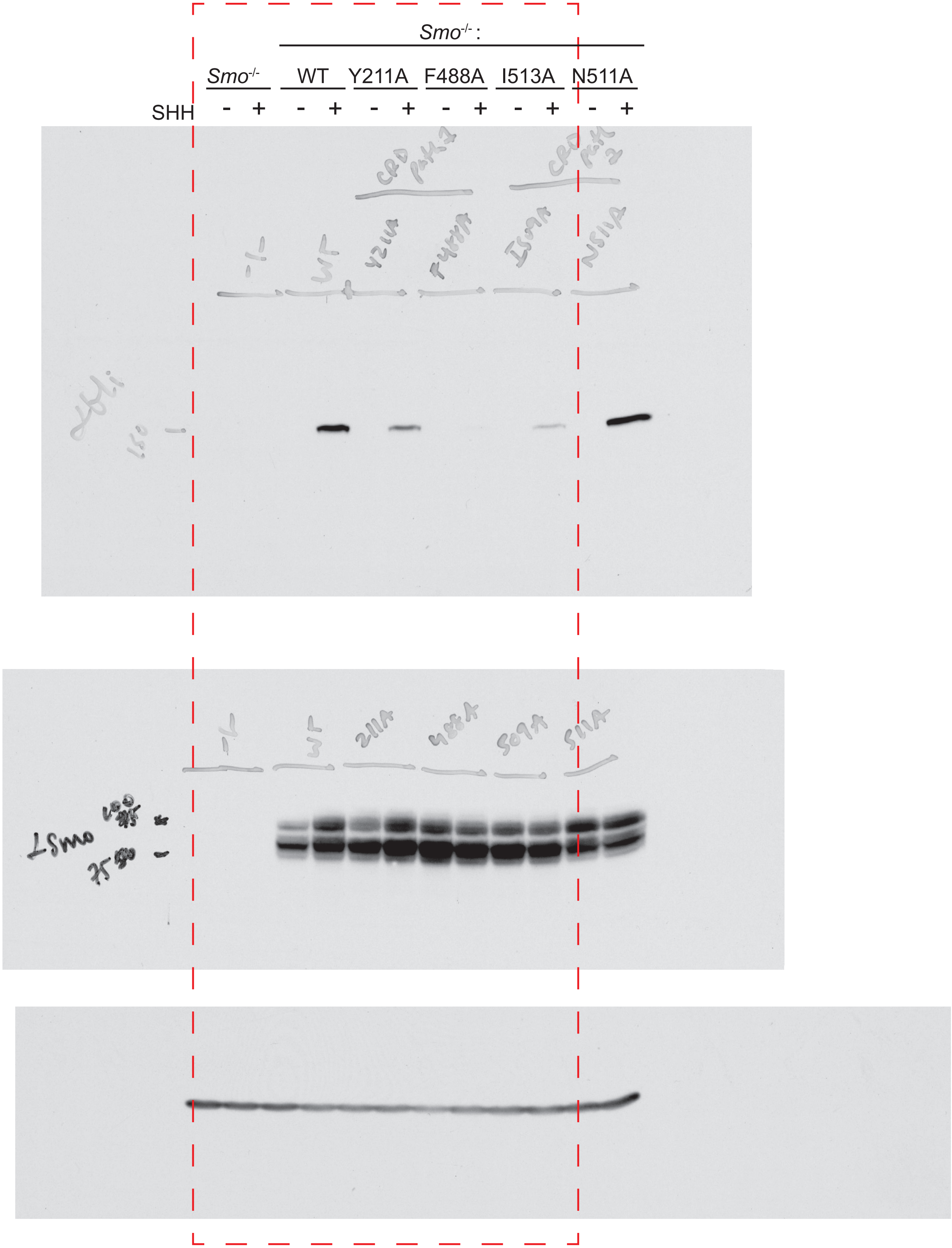

Supplement: Figure 6—figure supplement 1—source data 1. [file elife-108030-fig6-figsupp1-data1.zip › Fig6_FigSupp1a_original_pdf.pdf]
